# ﻿*Naviculacongqiancuoensis* sp. nov. — a new diatom species (Bacillariophyceae) from Sichuan Province, China

**DOI:** 10.3897/phytokeys.257.152987

**Published:** 2025-06-11

**Authors:** Yu-Fei Jiang, Saúl Blanco, Wei Zhang

**Affiliations:** 1 Key Laboratory of Exploration and Utilization of Aquatic Genetic Resources (Ministry of Education, China), Shanghai Ocean University, Shanghai 201306, China Shanghai Ocean University Shanghai China; 2 Diatom Laboratory, University of León, La Serna St., 58, 24007 León, Spain University of León León Spain

**Keywords:** China, Lake Congqiancuo, *Navicula*, new species, taxonomy

## Abstract

In this study, we describe a new diatom, *Naviculacongqiancuoensis***sp. nov.**, which was found in epilithon samples collected from a small mountain lake, at Lake Congqiancuo, Sichuan Province, China. A detailed morphological description of the new species is provided based on light and scanning electron micrographs. *Naviculacongqiancuoensis***sp. nov.** valve is linear to linear-lanceolate, with a slight elevation in the center and broadly rounded at both apices. The striae are radiate and convergent at the apices. The axial area is narrow and linear, and the central area is small and elliptical in the middle. This new species is compared with similar species, such as *Naviculaangusta*, *N.leptostriata*, *N.piercei*, *N.heimansioides* which differ in size, valve shape, striae density and ultrastructure.

## ﻿Introduction

The genus *Navicula* Bory (1822), initially described by Bory de Saint-Vincent in 1822, has undergone several revisions over time, including those by Patrick (1959), Cox (1979), and Round et al. (1990). Since its original description, numerous genera have been separated from *Navicula*, such as *Diadesmis*Kützing (1844), *Luticola* D.G.Mann in Round et al. (1990), *Gandhia*Kulikovskiy et al. (2023), *Trialacinia* Kociolek, Liu & Fan (2025). Consequently, the current definition of *Navicula* sensu stricto is based on the type of *Naviculatripunctata* (Müller 1786) Bory (1822), and is restricted to the members of the section Lineolatae previously described by Cleve (1895) as Cox (1979). Species within the genus *Navicula* typically exhibit a narrow to broad lanceolate, naviculoid valve outline with apices that display a variety of shapes. The central sternum is asymmetrically thickened to varying degrees. The raphe branches are straight and filiform, with the proximal ends slightly deflected unilaterally and the distal ends strongly recurved. The striae are uniseriate and composed of apically elongated areolae called lineolae, characteristics that facilitate the identification of *Navicula* among other diatom genera (Lange-Bertalot 2001).

To date, according to Diatom base, > 1000 species of the genus *Navicula* have been accepted taxonomically (Kociolek et al. 2024). Despite this, many *Navicula* species in inland waters remain undescribed, with numerous new species discovered globally over the past decades. These discoveries span Africa, Europe, America, Asia, and even Antarctica (Taylor et al. 2016; Van de Vijver and Lange-Bertalot 2009; Witkowski et al. 2010; Edlund and Soninkhishig 2009; Zidarova et al. 2016).

In China, the genus *Navicula* is still insufficiently studied. Li and Qi (2016) listed 133 species and 32 varieties in the Diatom Flora of China, yet it is evident that an in-depth study of this genus within the country’s borders is still lacking. Recent findings by Gong et al. (2015) of new species from the Yunnan Plateau’s lakes include *Naviculacraticuloides* Y. Li & Metzeltin, *Naviculagongii* Metzeltin & Y. Li and *Naviculayunnanensis* Y. Li & Metzeltin. Adding to this diversity, Chudaev and Georgiev (2016) added two new Navicula in a high-altitude Tibetan lake: *Naviculagololobovae* Chudaev in Chudaev and Georgiev (2016) and Naviculacryptofallaxvar.tibetica Chudaev in Chudaev and Georgiev (2016). Meanwhile, in a stark contrast of habitat, a new benthic brackish diatom species, *Naviculaamoyensis*, was identified in the estuarine environment of the Jiulong River (Chen et al. 2017) in Southern China. Furthermore, expanding the known diatom flora of China, Fu (2018) documented six previously unrecorded *Navicula* species from the Changbai Mountain regions. Wang et al. (2020) reported a new species, *Naviculadaochengensis*, from Lake Congqiancuo in Sichuan, China, highlighting the ongoing exploration and documentation of *Navicula* diversity in the country. Furthermore, *Naviculaaustralasiatica*, *Naviculaperangustissima* and *Naviculaturriformis* in Li et al. (2020); *Naviculafuxianturriformis* in Zhang et al. (2022) and *Naviculasinicomeniscus* in Xiao et al. (2023).

The aim of this paper is to describe a new *Navicula* species, *Naviculacongqiancuoensis* sp. nov., from a small mountain lake — Lake Congqiancuo, Haizishan Nature Reserve, Daocheng County (Sichuan Province, China) — based on its unique morphological features, revealed by detailed light and scanning electron microscopy (SEM) observations and its comparison with similar taxa.

## ﻿Material and methods

### ﻿Study area

Samples containing *Naviculacongqiancuoensis* sp. nov. were collected from pebbles (i.e. epilithon) in the littoral zone of Lake Congqiancuo, located at the geographic coordinates 29°21'N and 100°04'E. The lake is situated within the Haizishan Nature Reserve, found in Daocheng County, Sichuan Province, China, in the southeastern part of the Qinghai-Tibet Plateau. The elevation within the area where Lake Congqiancuo is located ranges from 3160 to 6204 m a.s.l., with an average altitude of around 4200 m. The region experiences a highland climate, characterized by an annual mean temperature of approximately 3.0 °C and an average annual precipitation of roughly 720 mm. Previously, we reported the discovery of two new diatom species, *Halamphoradaochengensis* W. Zhang, Jüttner & Levkov and *Naviculadaochengensis* W. Zhang, Chudaev & T. Wang, from this freshwater lake. However, it remains largely under-investigated with the likelihood that numerous diatom species are yet to be identified and studied.

### ﻿Field and laboratory procedures

Water physico-chemical characteristics were measured using a YSI Pro Plus multimeter, and Secchi Depth was determined following APHA’s standard procedures (Odum et al. 1995). Chemical analyses samples (1 L water) were collected from surface waters (the top 50 cm) at 12 sampling points (Zhang 1991), including total phosphorus (TP), total nitrogen (TN). Samples were initially fixed in 10% Lugol’s solution. In the laboratory, 10% hydrochloric acid was used to dissolve the carbonate content, and 30% hydrogen peroxide was applied to oxidize the organic components of diatom cells, following the protocol described by Battarbee (1986). After the digestion process, which included heating at 80 °C for 6 hours, the samples underwent centrifugation (3500 rpm for 5 min) and multiple washes with distilled water (4–5 times) until the pH reached neutrality. The cleaned material was stored in 95% ethanol. Permanent mounts were prepared using Naphrax for examination under an Olympus BX53–DIC compound microscope equipped with an Olympus DP72 digital camera. Additionally, the cleaned material was observed using a Hitachi S–3400N field-emission scanning electron microscope (SEM) at Shanghai Ocean University (operating voltage 5 kV). All samples and slides are deposited in the Herbarium of Hydrobiological Department, Shanghai Ocean University (SHOU), China.

## ﻿Results

**Class Bacillariophyceae**

**Order Naviculales**

**Family Naviculaceae**

**Genus *Navicula***

### ﻿ Navicula congqiancuoensis

Taxon classificationPlantaeNaviculalesNaviculaceae

W.Zhang & S.Blanco sp. nov.

459382DC-D60A-5C43-9554-60E07D5A482F

LM: Figs 1–7 , SEM: Figs 8–13 , 14–18

#### Description.

**Figures 1–7.**
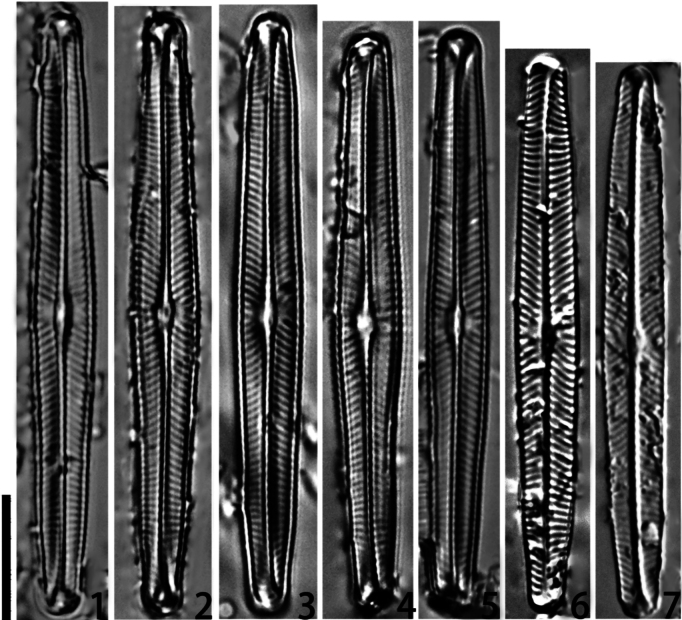
*Naviculacongqiancuoensis* sp. nov. Size diminution series. Scale bar: 10 μm.

***Light microscopy*** (LM) (Figs 1–7). The valve is linear to linear-lanceolate, with a slight widening in the center and broadly rounded at the apices, with a length of 42.7–51.2 μm and a width of 4.9–6.1 μm, giving an average L/W ratio 8.2 (40 valves were measured to obtain these size ranges). The raphe is weakly lateral, and the external distal raphe ends are deflected on to the mantle. The striae are radiate and convergent at the ends, and there are two to three short striae in the central area, 14–16 in 10 μm. The axial area is narrow and linear, and the central area is small and elliptical in the central nodule.

**Figures 8–13.**
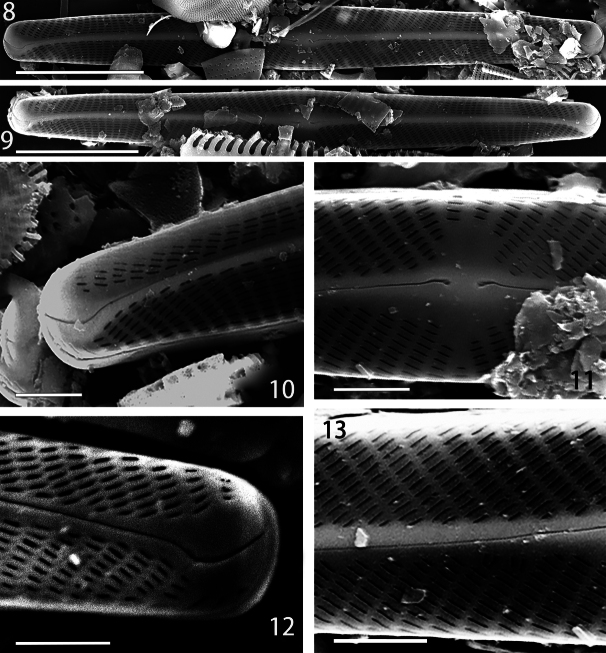
*Naviculacongqiancuoensis* sp. nov. SEM external views **8, 9** whole valve showing the outline and external raphe structure **10, 12** distal raphe ends and areolae **11** details of external central area with slightly expanded proximal raphe ends **13** details of external view of striae. Scale bars: 10 μm (**8, 9**); 2 μm (**10, 11, 12, 13**).

**Figures 14–18.**
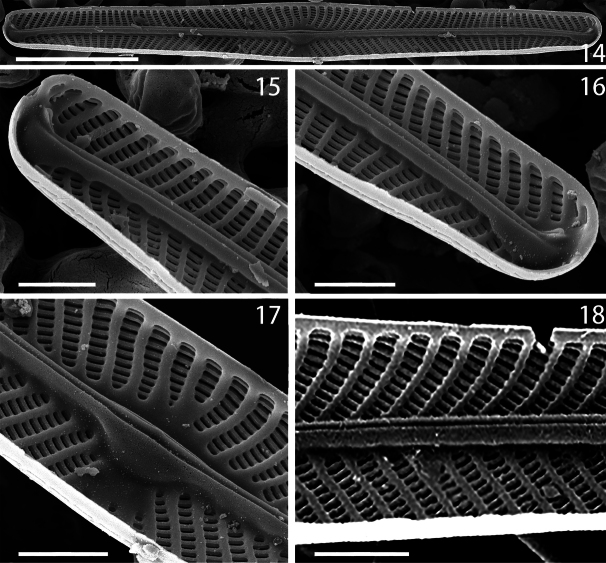
*Naviculacongqiancuoensis* sp. nov. SEM internal views **14** whole valve showing the outline and internal raphe structure **15, 16** distal raphe endings with helictoglossae **17** central area **18** details of areolae. Scale bars: 10 μm (**14**); 2 μm (**15, 16, 17, 18**).

***Scanning electron microscopy*** (SEM) (Figs 8–18). External view: Valves flat. Raphe fissure straight (Figs 8, 9). Proximal raphe endings slightly unilaterally hooked towards secondary valve side. External distal raphe endings curved to the secondary side of valve, with ends positioned on valve mantle (Figs 10, 12). Axial area very narrow and often thickened. Lineolae density *ca.* 50 in 10 µm (Fig. 11). Transversal striae radial, convergent at the ends. The lineolae and the virgae separating them are aligned longitudinally so that there appear to be both longitudinal and transverse striae (Figs 8–13). Internal view: the raphe is straight and slightly inclined towards the secondary valve side (Fig. 14). On the side of the raphe sternum opposite to central nodule, there is a longitudinal, semilanceolate thickening. Distal raphe endings are well-developed helictoglossae (Figs 15, 16). The accessory rib is well-developed on the primary valve side and unilaterally widened in the center, forming an asymmetrical central nodule that is visible under light microscopy (Figs 17, 18). The Voigt discordance is not visible in the valves we observed.

#### Type.

China. • Sichuan Province: Tibetan Autonomous Prefecture of Garzi, Daocheng County, Haizishan Nature Reserve, Lake Congqiancuo, 29°21'58"N, 100°04'51"E, elevation 4389 m a.s.l., samples collected by Q.C. ZHOU, 20 Sep. 2018. Holotype CQC1#, SHOU! Herbarium of Hydrobiological Department, College of Fisheries and Life Science, Shanghai Ocean University (SHOU), Shanghai, China = Fig. 3.

#### Etymology.

The specific epithet, *congqiancuoensis*, refers to the locality from which the new species was described.

#### Ecology.

*Naviculacongqiancuoensis* sp. nov. was found in epilithon samples collected in a high-altitude freshwater lake, with a pH of 8.9–9.4 and very low conductivity of 17.5–17.6 µS/cm. The water temperature at the time of collection was 13.5 °C, total nitrogen (TN) 0.032–0.043 mg/L, total phosphorus (TP) 0.014–0.016 mg/L.

## ﻿Discussion

*Naviculacongqiancuoensis* sp. nov. shares some features with other taxa in this genus, such as *Naviculaangusta*Grunow (1860), *N.leptostriata*Jørgensen (1948) and *N.piercei*Bahls (2012). They all are more or less similar in valve outline (linear or linear-lanceolate), raphe (almost lateral) and axial area (narrow and linear). A comparison of characteristics of these taxa in *Navicula* is provided in Table 1.

**Table 1.** Morphological characteristics of *Naviculacongqiancuoensis* sp. nov. and morphologically related species.

	*N.congqiancuoensis* sp. nov.	*N.angusta*Grunow (1860: 528)	*N.leptostriata*Jørgensen (1948: 59)	*N.piercei*Bahls (2012: 28)	*N.heimansioides*Lange–Bertalot (1993: 113)
Outline	Linear to linear-lanceolate	Linear	Narrowly lanceolate	Linear and subtly triundulate with a distinctly tumid middle	Lanceolate to linear-lanceolate
Ends	Broadly rounded	Slightly protracted ends	Acutely rounded, subtly protracted apices	Broadly rounded, somewhat wedge-shaped apices	Slightly drawn-out ends
Axial area	Narrow and linear	Narrow	Narrow	Very narrow and linear	Narrow
Central area	Slightly widening	Moderately asymmetrical, wedge-shaped or irregularly shaped	Small, transversely widened, and asymmetrical with irregular borders.	Elliptical and asymmetric, bordered by 2–5 irregularly shortened and often faint striae.	Small
Valve length (μm)	42.7–51.2	42.0–58.0	33.0–39.0	64.0–76.0	18.7–39.3
Valve width (μm)	4.9–6.1	6.0–7.0	4.9–5.7	6.8–8.0	5–6
Striae in 10 μm	18–20	12–13	19–20	11–13	14–16
Areolae in 10 μm	50	32	Not given	30	Not given
Distribution	Lake Congqiancuo, Haizishan Nature Reserve, Daocheng County, Sichuan Province, China	Widely distributed	Northern Rocky Mountains, where it is typically associated with Navicula notha.	Copper Lake in Shoshone County, northern Idaho	Widely distributed
Reference	This study	Grunow 1860, Lange-Bertalot 1996	Jørgensen 1948, Lange-Bertalot H 2001	Bahls 2012	Lange–Bertalot 1993, Taylor 2016

*Naviculacongqiancuoensis* and *N.piercei* (Bahls 2012) exhibit similarities in their valve shapes, yet there are subtle distinctions. Both possess linear valves; however, in *N.piercei* they are distinctly tumid in the middle and with broadly rounded, somewhat wedge-shaped apices, a feature not present in *N.congqiancuoensis*, which is also shorter compared to *N.piercei* (42.7–51.2 μm *vs.* 64–76 μm). Additionally, the stria density is higher in *N.congqiancuoensis* than in *N.piercei* (14–16 in 10 µm *vs.* 11–13 in 10 µm). Furthermore, the areola density of *N.congqiancuoensis* is higher than that of *N.piercei* (50 areolae in 10 μm *vs.* 30 areolae in 10 µm).*Naviculacongqiancuoensis* and *N.angusta* (Grunow 1860) exhibit certain differences in their valves: in *N.congqiancuoensis* is linear to linear-lanceolate, with a slight widening in the center and broadly rounded ends, while in *N.angusta* valves are linear with slightly protracted ends. *Naviculacongqiancuoensis* is also narrower than *N.angusta* (width: 4.9–6.0 μm *vs.* 6–7 μm). The stria density of *N.congqiancuoensis* is higher than that of *N.angusta* (15–18 in 10 µm *vs.* 12–13 in 10 µm). Furthermore, the areola density of *N.congqiancuoensis* is higher than that of *N.angusta* (50 areolae in 10 μm *vs.* 32 areolae in 10 µm). *Naviculacongqiancuoensis* and *N.leptostriata* (Jørgensen 1948) show also certain differences in their valves: *N.congqiancuoensis* has linear to linear-lanceolate valves, with a slight widening in the center and broadly rounded ends, while in *N.leptostriata* valves are narrowly lanceolate with acutely rounded, subtly protracted apices. *Naviculacongqiancuoensis* is also longer *N.leptostriata* in length (42.7–51.2 μm *vs.* 33–39 μm), and the stria density is lower in *N.congqiancuoensis* compared to *N.leptostriata* (15–18 in 10 µm *vs.* 19–20 in 10 µm). *N.congqiancuoensis* is longer than *N.heimansioides* (length: 42.7–51.2 μm *vs.* 18.7–39.3 μm) (Lange–Bertalot 1993). And *N.heimansioides* valves are lanceolate to linear-lanceolate with slightly drawn-out ends, *N.congqiancuoensis* exhibit linear to linear-lanceolate valve shapes with broadly rounded ends. The stria density of *N.congqiancuoensis* and *N.heimansioides* are the same (14–16 in 10 µm), and both striae are radiate and bent at the valve center, convergent at apices.

To sum up, the discovery of *N.congqiancuoensis* in Lake Congqiancuo underscores the importance of continued exploration of diatom diversity in remote and understudied regions. As we expand our knowledge of diatom taxonomy in China, findings such as *N.congqiancuoensis* contribute to a more comprehensive understanding of biodiversity patterns and ecological dynamics in high-altitude aquatic environments. Further research into the ecology and distribution of this species may provide valuable insights into the biogeography and environmental preferences of diatoms in mountainous freshwater systems.

## Supplementary Material

XML Treatment for Navicula congqiancuoensis

## ﻿Additional information

### Author ORCIDs

Yu-Fei Jiang https://orcid.org/0009-0003-9099-9697

Saúl Blanco https://orcid.org/0000-0002-9015-2512

Wei Zhang https://orcid.org/0000-0002-8626-6138

### Data availability

All of the data that support the findings of this study are available in the main text.
